# Answering Big Questions in Pain Medicine

**DOI:** 10.7759/cureus.43561

**Published:** 2023-08-16

**Authors:** Antonella Paladini, Ricardo Vallejo, Marixa Guerrero, Alberto Pasqualucci, John F Peppin, Joseph Pergolizzi, Giustino Varrassi

**Affiliations:** 1 Department of Life, Health & Environmental Sciences (MESVA), University of L'Aquila, L'Aquila, ITA; 2 Department of Research, Millennium Pain Center, Bloomington, USA; 3 Department of Pain Medicine/ Pain Management, Clínica del Country, Bogota, COL; 4 Department of Anesthesia and Critical Care, University of Perugia, Perugia, ITA; 5 Department of Osteopathic Medicine, Marian University, Indianapolis, USA; 6 Department of Anesthesiology, Pain Medicine, and Critical Care Medicine, Nema Research, Naples, USA; 7 Department of Pain Medicine, Paolo Procacci Foundation, Rome, ITA

**Keywords:** histones, ephrins, sigma receptors, analgesic targets, opioid use disorder, opioids, pain medicine, analgesia

## Abstract

The future of pain medicine is marked by many questions. What can other nations around the world learn from the opioid crisis that is still affecting the United States? The American opioid experience was mischaracterized and wrongly described, and its causes were misdiagnosed from the outset, leading to its mismanagement and the abandonment of many chronic pain patients to their suffering. There are a few new drugs in the analgesic armamentarium. What new targets do we have in pain medicine? There are many breakthroughs, discoveries, and potential new targets that could add to our analgesic prescribing choices. These include sigma receptors, d-amino acid oxidase, endoplasmic reticulum stress receptors, histone deacetylase, and others. Neuromodulation had been used with varying degrees of success for years, but with a simplistic approach based on the gate theory of pain. Despite our familiarity with neuromodulation and spinal cord stimulators, neuromodulation research indicates that the activation of glial cells may activate the immune system and enhance analgesia. Neuromodulation studies have concentrated on how electricity affects neuronal activity rather than how electrical activity could reduce pain. There are still more frontiers in our battle against pain and some promising avenues for treatments. This narrative review will try to summarize what can be done from the perspective of recent technological and pharmacological developments.

## Introduction and background

When looking at the future of pain medicine, a short-range and a long-range view are necessary. In the short term, the opioid crisis in the United States has had a profound and adverse impact on pain treatment around the world. Chronic pain patients today may struggle to find adequate pharmacologic remedies, and to an extent, some clinicians may feel that there are few or perhaps no real answers for certain painful conditions. It seems as if there have been no recent breakthroughs in pain care, only setbacks.

In the long run, there is much to hope for in pain medicine. The growing understanding of the underlying mechanisms of pain has opened a wealth of new potential therapeutic targets for drug development. Neuromodulation, which was once viewed simplistically as a device-based approach based on the gate theory of pain, may offer benefits based on the restoration of a neuronal-glial balance that activates the immune system. Greater elucidation of the neuronal and glial systems has led to a new appreciation and perhaps new applications of neuromodulation, especially in the form of direct, targeted multiplexer simulation models. There is much to look forward to in our ongoing medical efforts to conquer pain, although many painful conditions remain challenging to treat.

This review is based on presentations and expert discussions presented at a scientific meeting in Cancun, Mexico, in April 2023. The presentations were based on a review of the literature and real-world clinical practice. The aim was to present an overview of some of the biggest questions facing pain medicine today. This narrative review article aims to summarize the treated topics and open new frontiers in the management of pain patients.

## Review

What can be learned from the U.S. experience with opioids?

Value of Death Certificates and Their Role in the "Opioid Crisis"

The U.S. public health crisis surrounding opioids has impacted the entire world and has been largely defined by data obtained from death certificates. Despite the incredible weight these data have on our assessment of the crisis, they are fraught with errors. In one study, 82% of American death certificates were found to contain errors [1]. Another study found that 46.5% of those filling out death certificates knowingly recorded false information, and only 33% of those filling out the forms believed that the correct cause of death was entered [2]. In another study, only 15.5% of those completing a death certificate were able to accurately name the cause of death [3]. Even the National Vital Statistics Services (NVSS) of the Centers for Disease Control and Prevention (CDC) stated that one out of every three death certificates contained errors and that such high error rates would rather increase than decrease. This statement was made just before the COVID pandemic, which further challenged death certificate accuracy [4]. This problem is not unique to the United States: 32.9% of death certificates in Canada had errors [5]; 78.4% in Nepal [6]; 30% in Peru [7]; and in Taiwan, only 61% of death certificates were correctly prepared [8].

Death certificate data also present other problems. In Italy, as in some parts of the United States, there is no official organization to investigate deaths, such as a coroner’s office, leaving untrained clinicians to complete death certificates [9]. This work may be completed based on an external examination, limited or no review of medical records, circumstantial findings, and no autopsy [10].

In the United States, an overdose victim is typically brought to an emergency department, where the physician or other healthcare professional completes the death certificate, or in other cases, the funeral director handles the paperwork. In both cases, clinicians or funeral home staff have considerable discretion in this task and may bring their own biases to bear. In the same country, a local medical examiner or the coroner’s office may see fit to examine the death certificate, but this step is optional. Data from the death certificate are first entered into the state database, whereupon data are taken up by the federal and World Health Organization (WHO) databases [11]. Every step in this ponderous chain has the potential to introduce errors.

Toxicology reports are sometimes used to help determine the cause of death, but lethal drug concentrations can have broad ranges. Individuals with chronic opioid use disorder may have built up a tolerance to the point that they can survive with allegedly "toxic" drug concentration levels. Thus, the discovery of toxic concentrations of drugs is not necessarily proof of overdose death. With the emergence of more polysubstance overdoses and new designer drugs, not all substances may even appear in a standard toxicology screen.

While the high rate of errors on death certificates has been known for decades, this does not refute the notion of an opioid crisis. In the United States, chronic pain has historically been approached simplistically. Lack of reimbursement limits multimodal and multidisciplinary pain approaches and leads to reliance on oral opioids. People with opioid use disorder in the USA often lack access to safe, effective, and affordable rehabilitation. All of these things have contributed to the crisis, but not to our proper understanding of the role of opioids in pain medicine.

Mortality data often do not differentiate between deaths from prescription opioids versus deaths from illicit opioids, which tends to foster the notion that all opioid deaths are caused by people who obtain prescription pharmaceutical products from a physician for a legitimate indication. The CDC issued prescribing guidance to family practice physicians in an effort to reduce opioid deaths, but they recommended guidance only for prescription products administered under medical supervision rather than illicit drugs [12]. Thus, the data lumped together street drug users taking illicit opioids with pain patients taking prescription products under medical supervision. Guidance focused on clinicians and pain patients when, in fact, the opioid crisis was being driven by street drugs, illicit fentanyl, and the recreational use of polysubstance [13, 14]. People struggling with severe painful conditions were tapered off opioids or discontinued [15], while mortality data continued to climb. By vastly restricting pain treatments, the CDC launched a second health crisis, namely the undertreatment or, in some cases, the outright rejection of about 18 million people with moderate to severe chronic pain syndromes [16]. About three-quarters of all opioid deaths in the United States are caused by the use of illicit opioids, something the CDC guidelines for family practice physicians did not even mention [16]. The gravitas of the CDC led many healthcare institutions and healthcare professionals to regard these recommendations as quasi-law, even though they ran contrary to the real-world clinical experiences of clinicians in real-world practice.

Misdiagnosis by Various Agencies

Regulatory agencies and public health authorities have misdiagnosed the opioid crisis, which led to wrong decisions regarding how to address it. The media has been irresponsible in its reporting on this major crisis [17]. Today, some physicians simply will not accept chronic pain patients, and some have stopped treating these patients with opioids by abruptly discontinuing the opioid rather than offering a medically sound and sensible tapering program [15]. Some physicians will not prescribe opioids at all but refer patients to specialty pain clinics. In fact, the "non-interventional pain physician" is gradually becoming extinct in the United States. Many pharmaceutical companies have abandoned the pain space since new products are considered very risky, even if they are not opioids. Chronic pain patients, many of whom did well on long-term opioid therapy, were abandoned and left with few resources to manage their pain. Suicide rates in this population have increased [18]. By misdiagnosing the original crisis as one of overuse of prescription drugs in clinical settings, the original crisis of illicit drug use rages on, and a new crisis of untreated chronic pain has begun.

The crisis was exacerbated by our limited understanding of pain itself. Categorizing pain by type initially created a misleading binary concept of pain as either nociceptive or neuropathic [19]. In 2012, the International Association for the Study of Pain (IASP) introduced "nociplastic" pain [20] to expand the old model. These definitions and classifications omit a discussion of the most useful type of pain category for clinical practice: mixed pain. In primary care practices, mixed pain syndromes are frequently observed [19], for example, cancer pain, low back pain, and osteoarthritis. Treating one type of pain in a patient with a mixed pain syndrome can be only partially effective, at most.

Pain Management (and Opioid Use) in Elderly Patients

The elderly have disproportionately high rates of chronic pain, estimated at 25% to 80% [21], yet according to the Beers Criteria, many analgesics and adjunctive drugs are not suitable for them. In broad classes, these unsuitable drugs include muscle relaxants, nonsteroidal anti-inflammatory drugs (NSAIDs), tricyclic antidepressants, selective serotonin reuptake inhibitors, selective norepinephrine reuptake inhibitors, gabapentinoids in general but especially concurrently with opioids, and benzodiazepines [22]. With a long list of drugs either outright contraindicated or not recommended for seniors, there is a dearth of pain relievers available for geriatric chronic pain patients. From a meta-analysis, there is uncertainty about the safety and efficacy of muscle relaxants, which have been shown to not reduce pain but increase the risk of an adverse event [23]. Benzodiazepines have no valid role in pain treatment, although they are widely prescribed and may be considered overused [24]. Acetaminophen (paracetamol) has little evidence supporting its role as a chronic pain reliever [25]. Like NSAIDs, acetaminophen can increase blood pressure, and formulations with sodium increase cardiovascular risk, a particular concern for the geriatric population [26]. In addition, acetaminophen is not an adequate analgesic agent for certain painful conditions, such as postoperative pain, although it is sometimes administered in the United States in this way.

Pain Management Through Other Psychedelics

Despite the popularity of cannabinoids as a new type of pain reliever, many of the systematic reviews and meta-analyses about these agents do not provide a sufficient evidentiary foundation for reaching clinical decisions [27]. Results have been mixed, and harms associated with cannabis and cannabis-based medicine are a growing area of investigation [28]. In the United States, cannabis-related products are subject to a convoluted and murky legal status in that marijuana and related cannabinoid products are illegal at the federal level, but some states allow them for specific medical, and sometimes recreational, use.

Long-term data and evidence in the literature on interventional pain treatments for seniors are lacking. A meta-analysis found low-certainty evidence that spinal cord stimulators offered clinically meaningful pain relief [29]. Implantable intrathecal pumps for targeted drug delivery to the spine have not been studied in well-designed randomized clinical trials [30]. Seniors may be too frail for certain interventional therapies.

In pain medicine, reducing pain by 30% in a patient is often considered a treatment "success." Nowhere else in medicine is the bar set so low. Yet prescribers have few pharmaceutical options that can offer even 30% pain relief, and that includes opioid analgesics, which have well-known side effects that can be treatment-limiting. What is known about chronic pain is incomplete, and much of what we once "knew" turned out to be wrong or misleading. It is a sad but fair statement to say that physicians today do not know the optimal treatments and best practices for managing pain, especially chronic pain, and most especially chronic pain in geriatric patients.

While there is growing interest in the potential use of psychedelics for pain care, the literature and research in this field for their use in the context of chronic pain is limited [31], and realistically, solid evidence sufficient to guide clinical practice is years away. These agents include but are not limited to phenethylamines (mescaline), tryptamines (psilocybin), and ergolines (lysergic acid diethylamide (LSD)). Nanotechnology is of interest but likewise remains years away from any specific application [32].

Calcitonin gene-related peptide (CGRP) receptor antagonists, currently approved for migraines, have opened new possibilities for pain care, particularly in areas such as chronic visceral pain, pain associated with cardiac ischemia, somatic pain, COVID-induced lung inflammation, trigeminal neuralgia, peripheral neuropathy, and other difficult-to-treat painful conditions. Sodium-channel blockers are oral medications that may be used for acute pain, and phase 3 studies for the treatment of diabetic peripheral neuropathy are underway. The drawback to these agents is that they remain far in the future; realistically, it could be years before they can be used for chronic pain [33].

Pain Management and Economic Disparities

An important consideration in pain medicine is medical debt, which in the United States can be unpredictable, very high, and impacts low-income communities most severely. Low-income communities are also the most affected by chronic pain. Furthermore, many patients presenting with moderate to severe pain, particularly chronic pain syndromes, may not be believed by clinicians. The call to discontinue opioids is often perceived by pain patients as perplexing, particularly if they have found those drugs safe and effective over the course of years. The requirement to discontinue an effective therapy seems medically unsound. When patients are told they must begin a tapering regimen, they may feel stigmatized, targeted, and devalued; it also undermines their faith in their physicians and medical establishments [34].

The international pain community can learn from the American experience and not allow poorly written death certificates and a media narrative to define the opioid crisis. Extremes in care must be avoided; treating everyone with opioids was as damaging as treating no one with opioids. Pain cannot be viewed simplistically; it often presents in mixed pain syndromes that require multimodal and even multidisciplinary approaches.

Pain is a universal human experience, affecting about 20% of the world at any given point. Pain has been called the "hidden epidemic" [35]. The inability to obtain adequate analgesia has been associated with a two- to three-fold increase in suicide [18]. Typical chronic pain patients in the United States are unemployed or underemployed, sometimes due to disability. As a result, they often lack health insurance or have limited coverage. They cannot afford expensive options to treat their pain, and if they are older, such remedies may be contraindicated. This leaves the question: What do they do? Abandoning pain patients is not an option.

First, our database systems should be improved, fortified, and utilized so that we have reliable and adequate data about opioid-related deaths. We must call out false or misleading opioid-related stories in the media. Where it applies, such as in the United States, the outmoded coroner’s office should be replaced with medical examiners, ideally forensic pathologists. Death certificates should only be completed by physicians with special training. Finally, guidelines, policies, and regulations must all be based on evidence, meaning reliable and up-to-date data.

Healthcare professionals at all levels and in all disciplines receive only nominal training in pain management [36, 37]. Primary-care physicians see large numbers of patients dealing with chronic pain but often lack confidence and knowledge in terms of how to treat that pain. Thanks to draconian legislation, media-generated opioid hysteria, and poor public health response, many physicians are reluctant to prescribe opioids at all [38]. Medical schools fall short as well, in that very few offer much training on pain, although it is a very common medical complaint, and almost no training in palliative care for patients at the end of life. Likewise, medical curricula neglect information on opioid use disorder, drug diversion, overdose rescue, opioid toxicity, and substance use disorders in general. All healthcare professionals should receive training on these topics.

Finding safe, effective solutions for our pain patients is challenging [39]. Social determinants, psychological factors, comorbidities, mental health conditions, age, and any number of other factors further complicate pain care. When treating patients, it may be important to find out what works for them, particularly if pain can be controlled by exercise, hot or cold therapy, relaxation techniques, chiropractic, massage therapy, or any number of other options. In some cases, patients may not think about pain control beyond a prescription. Educating them in positive, empowering ways may help reduce their pain and make them more in control of their condition.

What new targets do we have in pain medicine?

Neuropathic pain, an increasingly prevalent condition that is challenging to treat, is caused by somatosensory nervous system dysfunction [40]. It often arises in the burgeoning number of patients with metabolic disorders. There are few effective treatments available, some of which may be associated with treatment-limiting side effects. Using preclinical models, scientific explorations are underway to better understand the molecular mechanisms of somatosensory dysfunction. Analyses of these targets may lead to better therapeutic options to manage pain, improve function, and enhance patient well-being. When it comes to targets for agents to treat pain, we have an important list of new targets (Table 1).

**Table 1:** Despite the perceived lack of innovation in pain pharmacology, a list of new agents presents important new drug targets. 5-Ht: 5-hydroxytryptamine, also known as serotonin; NDMA: N-nitrosodimethylamine; AMPA: alpha-amino-3-hydroxy-5-methyl-4-isoxazolepropionic acid; GABA: gamma-aminobutyric acid; BKCa: big-conductance calcium-activated potassium channels; MAP: mitogen-activated protein; ERK: extracellular signal-regulated kinase; JNK: jun N-terminal kinase; TNF: tumor necrosis factor; IL: interleukin; CB1: cannabinoid receptor 1; CB2: cannabinoid receptor 2; PPAR: peroxisome proliferator-activated receptors; Na: sodium; Ca2+: calcium ion; CGRP: calcitonin gene-related peptide

Old Targets	New Targets
Noradrenaline and 5-HT transporter inhibitors	Sigma receptors
Voltage-gated sodium channels (Na(v)1.3, 1.7, 1.8, 1.9)	D-amino acid oxidase
Voltage-gated calcium channels (Ca(v)1.2, 2.2, 2.3, 3.1, 3.2 and α2δ subunits)	Endoplasmic reticulum stress receptors
Purinergic receptors (P1, P2Y, P2X)	Cyclic nucleotide-gated cation channels activated by hyperpolarization
Transient receptor potentials (TRPM8, TRPA1, TRPV1)	Histone deacetylase
Metabotropic (mGlutR1-mGluR5)	Wnt/β-catenin and Wnt/Ryk
Ionotropic (NMDA and AMPA) glutamate receptors and glial glutamate transport	Ephrins
Voltage-gated potassium channels (KATP, Kir6.2/SUR1, Kir6.2/SUR2, KCNQ (K(v)7.1-K(v) 7.5, and BKCa	Eph receptor tyrosine kinase
GABA receptors and transporters (GAT-1, GAT-3)	CDH1 and mitochondrial ATPase
MAP kinase (ERK, p38, and JNK MAP kinases)	
Pro-inflammatory mediators (TNF, IL-1, IL-6)	
Endocannabinoids (CB1 and CB2 receptors, fatty acid amide hydrolase, and transporters)	
PPAR-[26], Na+/Ca_2_+ exchanger	
Nitric oxide	
CGRP	
Neuronal nicotinic receptors	

Sigma Receptors

First described in 1960 but erroneously described as a subtype of opioid receptor, sigma (σ) receptors turned out to be a novel form of ligand-operated molecular chaperone [41, 42]. By 1994, two distinct subtypes were found, named σ1 and σ2 [43]. The first type was cloned in 1996 [44-46], but σ2 was not cloned until 2017 [47]. The first crystal structure of the σ1 receptor was described by Schmidt and colleagues in 2016 [48, 49].

The σ1 receptors are located in the mitochondrial-associated endoplasmic reticulum membrane (MAM), which is densely located in the heart, liver, nervous system, and immune cells [50]. These σ1 receptors are associated with pain, especially, but not limited to, neuropathic pain. In simple terms, a nerve injury activates the σ1 receptors, launching a series of physiologic processes resulting in neuropathic pain. The σ1 antagonists have been shown to reduce this pain in a dose-dependent fashion. The neural injury itself results in phosphorylation of the NR1 subunit of the N-methyl-D-aspartate (NMDA) receptor; it increases p47 phox, which increases nicotinamide adenine dinucleotide phosphate hydrogen (NADPH) oxidase 2 (Nox2) and results in an increase in reactive oxygen species. D-serine and its racemate increase, and calcium-dependent cascade or nitric oxide signaling pathways increase p38 mitogen-activated protein kinase (MAPK) activation [50]. Furthermore, σ1 receptor antagonists have been shown to stop the wind-up phenomenon in isolated spinal cords [50]. This has led to speculation that sigma ligand might also find applications in oncology and in the treatment of neurodegenerative disorders.

When activated, the σ1 receptor recruits immune cells and releases pain mediators by causing gliosis and decreasing microglia in the prefrontal cortex. This may be beneficial for osteoarthritis-associated pain. It also causes a series of downregulations in the spinal cord involving tumor necrosis factor (TNF), interleukin 18 (IL-18), astrocytes, and microglia. Tumor necrosis factor is associated with cancer pain and pain from osteoarthritis; IL-18 is associated with osteoarthritis pain and inflammatory pain conditions; astrocytes are associated with both peripheral and central neuropathic pain conditions as well as inflammatory pain; and microglia are associated with pain from osteoarthritis, cancer, and inflammation. Meanwhile, there is also an effect on the dorsal root ganglia, where the σ1 receptor activation downregulates IL-6, chemokine ligand 2 (CCL2), and macrophages, all of which are associated with neuropathic pain; it also downregulates brain-derived neurotropic factor (BNDF) associated with the pain from osteoarthritis. There is further activity in the inflamed tissue itself where the immune system releases opioid peptides [50].

The σ2 receptor was recognized as an important new target in oncology when it was found that σ2 receptor agonists could kill tumor cells using both apoptotic and non-apoptotic mechanisms in breast cancer, colon cancer, melanoma, and lung cancer. Not only that, σ2 receptors are 10 times as dense in the cells of proliferating tumors versus quiescent ones and are used as biomarkers for tumor growth. Selective ligands for σ2 receptors are being developed, and σ2 agonists may emerge as anticancer drugs. Note that the binding site of the σ2 receptor is localized within the progesterone receptor membrane component 1 (PGRMC1).

Many σ1 and σ2 receptor agonists and antagonists are in development [51-53]. Inhibition of the σ1 receptor prevents peripheral neuroinflammatory processes, such as macrophage infiltration of the dorsal root ganglia, while at the same time preventing central neuroinflammation, which involves activating the microglia and astrocytes. In early studies, it appears that such σ1 blockers enhance peripheral endogenous opioid analgesia driven by the immune system, optimizing the analgesic abilities of peripheral immune cells. For this reason, there is considerable interest in σ1 antagonists as a new class of analgesic agents that may offer a novel mechanism of action that could be of benefit in a variety of painful conditions.

Ephrins

Among the tyrosine kinase receptors, the erythropoietin-producing hepatocellular (Eph) family is the largest, most ubiquitous, and functionally varied receptor family in humans. Fourteen different Eph receptors have been identified in mammals, which can be grouped into two main receptor classes, A and B [54]. Via ligands that bind to the membranes known as Eph receptor-interacting proteins (ephrins), Eph receptors are associated with numerous immunologic and other functions from embryonic development through adulthood [55].

While EphA and EphB receptors are structurally similar, ephrinA and ephrinB ligands are markedly distinct in structure [55]. Bidirectional signaling between Eph receptor and ephrin ligands is a unique feature that occurs once the receptor binds to the ligand; there is also a characteristic clustering of Eph-receptor-expressing cells, sometimes called “forward signaling” [56, 57]. These receptors contribute to angiogenesis, axon guidance, and the creation of synapses. It is believed that EphB receptors and ephrins are responsible for synaptic activity modulation in the spinal cord. The ephrin system likewise plays a role in pathologic and physiologic pain modulation [55].

The Eph receptors and their ephrin ligands play a role in the embryonic development of the central nervous system, regulating cell or axonal migrations and synapse formations [58]. In adults, Eph receptors and ephrin ligands continue to play a neurologic role, this time in neuronal plasticity such as shaping dendritic spines [59]. These functions are only beginning to be elucidated and are made more complex in that both the Eph receptors and their corresponding ligands are expressed in pre-synaptic as well as post-synaptic neurons. The interactions between the "forward signaling" of the Eph receptors and the corresponding "reverse signaling" of the corresponding ephrin ligand are not entirely understood [60]. Activation of the signaling pathways between the EphB receptor and ephrinB in a murine model was not able to induce diabetic neuropathic pain but could sustain this pain syndrome once it started. In this murine study, blocking the EphB receptor could suppress diabetic neuropathic pain [61]. Nerve injury increases the expression of EphB and ephrinB, leading to a cascade of events that increase the excitability of nociceptive neurons and synaptic plasticity, leading to neuropathic pain.

Endoplasmic Reticulum Stress (ERS) Receptors

The endoplasmic reticulum is a large and dynamic network of tubules that resides in the cytoplasm of a eukaryotic cell. It has numerous functions, among the most important of which are calcium storage, lipid metabolism, and the synthesis of proteins. The endoplasmic reticulum is also tasked with folding proteins. When proteins are misfolded, endoplasmic reticulum stress (ERS) occurs, which interferes with normal cell functions. The response to ERS is the "unfolded protein response" (UPR). Endoplasmic reticulum stress can be triggered by fatty acids, insulin, oxidation stress, and the inflammatory response. However, ERS can also be caused by glucose deprivation, the depletion of calcium stores, and/or the presence of free radicals. When ERS occurs, it activates SREPB-1c, causes fatty acid synthesis, degrades apoB-100, and inhibits very low-density lipoprotein (VLDL) production, which increases fat accumulation in the liver. When ERS occurs, it increases the expression of blinding immunoglobulin proteins (BIP) [62-64]. 

Endoplasmic reticulum stress is associated with inflammatory diseases, particularly neuroinflammatory processes. Pro-inflammatory mediators can trigger the ERS, which can then in turn result in neuropathic pain [64]. The ERS response and autophagy are associated and may play a neuroprotective role. Certain σ1 receptor agonists may be helpful in initiating this ERS response and playing a beneficial role in neurodegenerative disorders such as Alzheimer’s disease, dementia, Parkinson’s disease, and motor neuron diseases [65].

Wnt/Catenin

The transmembrane proteins that mediate cell adhesion in animals are broadly known as cadherins. Cadherins support homeostasis and morphogenesis. The initial cadherin proteins discovered were epithelial cadherins (E-cadherins), neural cadherins (N-cadherins), and placental cadherins (P-cadherins). Note that N-cadherins are found in muscle and lens cells as well as neurons. When the cytoplasmic domains of various cadherins were studied, catenins, or specific and multifunctional proteins, were located. The β-catenin subunit helps to form adherens junctions that can create and maintain epithelial cell structures; these subunits also help form synapses and build neuronal circuits [66]. The enzyme receptor tyrosine kinase (RYK), a transmembrane protein tyrosine kinase, is necessary for axonal guidance and neuronal differentiation. Receptor tyrosine kinase has a binding affinity to Wnt [67, 68]. Wnt is a protein involved in cell fates such as apoptosis, axial polarity, and adhesion [69]. The signaling cascade of Wnt regulates development in animals and is a crucial driver of stem cell production in adult mammals, but mutations in these pathways may lead to cancer [70].

Multiple endocrine neoplasia type 1 (MEN-1) is an autosomal dominant form of cancer associated with parathyroid hyperplasia, pancreatic islet cell tumors, and anterior pituitary endocrine tumors, as well as other neoplasms [71]. The MEN-1 gene encodes for menin, a nuclear protein that may play a role in embryonic development as well. Menin plays a role in multiple diverse signaling pathways, and the depletion of menin can promote endocrine tumors. Menin likely plays a key role in the Wnt signaling processes of catenins, although the mechanisms are not elucidated [69]. Increased expression of catenins and menins in the dorsal spine is associated with hypersensitivity to pain, and disruptions in Wnt signaling with β-catenins have been linked to neuropathic pain and the release of pro-inflammatory cytokines [72]. Thus, β-catenin, Wnt/RYK, and Wnt-β-catenin make up an interesting new set of potential pharmacologic targets.

D-amino Acid Oxidase

D-amino acid oxidase (DAAO) is expressed in many animal species, but not in bacteria or plants. As an enzyme, DAAO works to oxidize D-amino acids into α-keto acids and, in so doing, produces ammonia and hydrogen peroxide [73]. In humans, DAAO is produced in the brain and has numerous physiologic effects, primarily on the brain. D-amino acid oxidase oxidizes D-amino acids from the flavin-adenine dinucleotide region (FAD) and is active in glial cells. D-amino acid oxidase produces D-serine amino acid, which increases synaptic activity at the NMDA receptors [74]. Since schizophrenia is associated with NMDA dysfunction and abnormally low levels of D-serine, it has been speculated that future treatments might include the administration of D-serine along with a DAAO inhibitor [75].

Injury or toxicity that damages a nerve can increase the expression and activity of DAAO within the spinal cord, which then activates the central astrocytes and, in turn, increases the production of reactive oxygen species. Reactive oxygen species can cause or contribute to neuropathic pain by directly or indirectly increasing the phosphorylation of NMDA receptors. Compared to the more active post-synaptic NMDA receptors, less is known about the pre-synaptic NMDA receptors, which may play a prominent role in neuropathic pain syndromes [76].

Histones

Histones are proteins found in the nuclei of eukaryotic cells, around which DNA winds itself like a thread on a spool to form a nucleosome. Nucleosomes, in turn, are densely packed together to form chromatin. Histones serve to protect DNA from damage and tangling and to reduce the size of long strands of DNA within the cell. When histones undergo acetylation, the electrostatic attraction between the histone and the DNA is weakened, and the chromatin structure is loosened. Acetylated histones are associated with active transcription. Acetylation has also been associated with neuropathic pain [77, 78].

Baicalin, a bioactive component derived from the herb *Scutellaria baicalensis*, can significantly attenuate the expression and suppress the symptoms of inflammatory pain [79, 80]. Valproic acid and suberoylanilide hydroxamic acid are deacetylase inhibitors, which can reduce pain [81, 82]. Curcumin is a polyphenol with potent anti-inflammatory action and can reduce pain together with the hypoacetylation of histones H3 and H4 in the dorsal ganglia [83]. Pretreating patients with an inhibitor of histone deacetylation may potentiate the analgesic effects of glutamate agonists, which has demonstrated the potential of acetylation for reducing pain. The role of histone deacetylase blockers in chronic pain patients remains to be further elucidated [84].

Mitochondrial ATPase

The mitochondria help to produce energy and play a role in cellular signaling, apoptosis, the production of reactive oxygen species, and calcium homeostasis. One role of mitochondria is to provide adenosine triphosphate (ATP) for a variety of cellular functions [85]. Neurodegeneration may occur when mitochondria shift from producing ATP to consuming it. Neuropathic pain is thought to involve derangement of both mitochondrial function and bioenergy production [86].

Chemotherapy drugs such as oxaliplatin and paclitaxel can cause nitration of the superoxide anion in the mitochondria, leading to the production of peroxynitrite [87]. This suggests that chemotherapy-induced neuropathic pain may be caused by mitochondrial toxicity. When a nerve is injured or damaged in some way, oxygen consumption increases, which leads to hypoxia, acidosis, diminished glycolytic reserves, and less energy, which, in turn, leads to neuropathic pain [50].

Neuromodulation in pain management

Neuromodulation remains an important part of our armamentarium against pain. But to understand the future of neuromodulation, one must review some of the past. The use of electric energy or electromagnetic fields in medicine is well known, but a potential new application arose in the potential treatment of leishmaniasis. Leishmaniasis is a tropical disease caused by a single-cell parasite transmitted by sand fly vectors. Leishmaniasis produces secreted acid phosphatases. The application of various types of electrical energy resulted in overall cell aggregation in a manner related to the frequency and polarity of the electrical energy. Cells tended to aggregate under the area of the electrode. Biphasic and anodic electrical fields promoted more than just aggregation but resulted instead in cell clumping, which could be taken as a sign of cellular stress. Secreted acid phosphatases produced by these cells often increase with electrical fields. At 10 kHz of energy, enzymatic activity was inhibited and secretion of acid phosphatases increased. When electrical fields were created, it caused parasites to migrate toward that focus, which might enhance therapeutic interventions, particularly for cutaneous forms of the disease [88].

In an animal study, it was found that when immune cells were sensitized, the immune challenge could potentiate the pro-inflammatory response, resulting in the exacerbation of the illness or pain. For example, corticosterone and laparotomy sensitized cells, but the immune challenge provoked certain symptoms of pain but not all. In an animal study, this was created by a "two-hit" model in which a tail shock provoked the release of glucocorticoids, and then exogenous glucocorticoids were administered. Either of these two events increased the release of pro-inflammatory cytokines and potentiated some, but not all, of the sickness behaviors [89]. The result of this study was that sometimes there are multiple factors involved in the creation of painful symptoms, and in some cases, one factor alone would not suffice to cause symptoms. This may help us answer the mysteries of complex regional pain syndrome (CRPS), which develops in some patients but not others.

The current thinking about the pharmacologic management of pain involves targeting a single ion channel or one receptor with the goal of solving that one portion of the "pain problem." The shortfall in this approach is that there are at least 200 genes that could be counted as highly related to painful symptoms and their severity, and there are at least 20 specific and different activation pathways. One patient does not necessarily present with one pain pathway or one pattern of pathway dysregulation [90]. Thus, it becomes impossible for one drug with one target to address the complexity of pain.

An older experimental study of pain evaluated the expression of glial fibrillary acidic protein (GFAP) both at baseline and after chronic constriction of the sciatic nerve. The investigation sought to determine whether GFAP expression could be modified by an NMDA receptor antagonist. Glial fibrillary acidic protein expression at baseline was influenced by an endogenous NMDA ligand, but astrocyte hypertrophy was both caused and maintained by continuous activation of the NMDA receptors. In fact, GFAP might have utility as a biomarker of neuronal function [91]. An NMDA receptor antagonist decreased pain in the murine model, but it reversed glial activation. The number of astrocytes was reduced drastically. However, neuromodulation as a science tends to focus exclusively on the depolarization of neurons, when we have had evidence for several decades that other factors likely contributed to analgesia.

A basic biological truth is that all living organisms, whether single-celled organisms or humans, have cells with a protective membrane that shields them from the proximal cells. Every cell thus has an electrical potential because of the components within the membrane and external to it. The application of electricity to the body will have some impact on the cells because it will modify the voltage. This electricity will be both orthodromic and antidromic as action potentials shift. The resting potential of an astrocyte differs from that of a neuron. Shining light into an animal’s eye alters the action potentials of neurons as well as those of astrocytes. The application of current will cause membrane depolarization, depending on the frequency, pulse width, intensity, and charge balance of the current [91]. Electrical stimulation cannot depolarize the glia but will result in the release of glial transmitters such as glutamate and adenosine. Further, this electrical stimulation will cause glia-to-glia communication cascades in the form of astrocytic calcium waves. Note that there is no glutamate or gamma-aminobutyric acid (GABA) without the presence of astrocytes. Glutamate can provoke calcium waves to be produced by astrocytes in a process described decades ago as "long-range glial signaling" [92].

Astrocytes cannot produce action potentials, and glutamate is a neurotransmitter. Yet glutamate provoked calcium waves, which can be considered a means of communication among astrocytes [92]. In this context, it is crucial to bear in mind that glial cells do not communicate with action potentials or electricity, which means that they operate differently and much more slowly than neurons. There can be multiple concurrent processes going on in the body at entirely different rates of speed and through various mechanisms. Neurons work in an almost linear fashion through synaptic connections, but astrocytes broadcast their signals. One could think that neurons are like landline telephones, while astrocytes work like cell phones [93]. Neurons make up just 15% of the total cells in the brain, and glia make up the balance. Long relegated to descriptions of being "housekeeping cells" or even just filler material, glia today are known to be able to detect and even to some degree regulate electrical energy coursing through the brain [93]. Pathological forms of chronic pain cannot occur without the involvement of glial cells, which mediate neuroinflammatory processes. Yet scientists had long overlooked the role of glial cells because they did not have fire action potentials, so no electrode recorded their activities. The role of glial cells in chronic pain syndrome was dismissed without further study. This is an important point in any discussion about neuromodulation, which, from its inception, focused erroneously on how to optimize neuronal responses rather than how to manage pain.

Considering the conventional model of a neuron transmitting a pain signal by the release of several neurotransmitters, it is common to overlook the adjacent microglial and astrocyte cells surrounding the synapse, which is sometimes called the "three-part synapse." The microglial cell and the astrocyte work together to surround and almost form a vacuum zone around the synapse. While the microglia and astrocytes are initially unaffected by a pain signal, ectopic action potentials trigger the release of glutamate and CGRP, which activate the NMDA receptor. At this point, the microglia have become pro-inflammatory, and the astrocytes reduce the expression of glutamate transport to the point that glutamatergic control is lost [94]. This may mark the transition to chronic pain.

Neuropathic pain is characterized by neuroinflammation at the site of the injured nerve(s). The inflammatory cascade at the site of injury results in the release of immune-active substances such as cytokines, chemokines, and other neurotransmitters [95]. In a highly neuroinflammatory state, the glial cells of the spinal cord become activated and modulate neurotransmission, although these glial cells do not conduct electricity themselves. The glial cells exist as microglia and macroglia, to which astrocytes and oligodendrocytes belong. Both microglia and astrocytes are involved in the development and potentiation of neuropathic pain. For example, the microglia release tumor necrosis factor-alpha (TNFα), IL-1β, and IL-6, all of which are associated with painful symptoms. Microglia propagate the neuroinflammatory response, recruit other microglia, and activate adjacent astrocytes [95].

Most analgesics pattern their pharmacological intervention to modulate the release and activity of various neurotransmitters. However, the reduction of electrical signals by exogenous electrical signals can also reduce pain [95]. Animal studies have shown that there is an upregulation of the immune response and a release of cytokines with low-frequency electrical stimulation [96]. Acute stimulation of the Aβ fibers activates spinal astrocytes, while a P2X7 receptor antagonist suppresses them. In animal studies, it was found that low-frequency signals modulate the genetic expressions relating to the immune response, inflammatory response, and synaptic signaling, which in all cases involved activation of glial cells [96].

Waveforms are defined by four characteristics of the electrical output pulse: amplitude, frequency, intensity, and duration. Differential targeted multiplex programming (DTMP) is a novel stimulation algorithm that modulates the interactions between neurons and glia by selectively treating these cells in a differential fashion [97]. Spinal cord stimulation with DTMP has been shown to influence the expression of glia-related genes and modulate these transcriptomes toward healthier levels [98].

Waveform parameters such as monophasic or biphasic, cathodic or anodic, and symmetrical or asymmetrical affected gene expression in an animal model of neuropathic pain. Thus, it may be concluded that specific types of spinal cord stimulation waveforms can modulate transcriptional pathways involved in chronic pain [99]. This suggests moving beyond the traditional paradigm of neuronal activation via a spinal cord stimulator toward activation of the immune system and pain signaling processes. While there is a tendency to equate charge or voltage with the "potency" of spinal cord stimulation, other waveform alterations, such as pulse amplitude value, can in some cases achieve similar results using less energy. Transcriptomic changes can be induced by spinal cord stimulation, some of which can activate microglia. As the role of microglia in chronic painful conditions is elucidated, this type of electrical activation takes on an intriguing and important role [100]. In other words, the application of electrical energy changes the epigenetics of the cell.

Neuropathic pain operates via a multiplicity of pathways, some of which involve MAP kinases and nuclear factor kappa-light-chain-enhancer of activated B cells (NF-κB) signaling, which trigger immune response factors and have the potential to phosphorylate numerous proteins, rendering them active or inactive. In an animal model of neuropathic pain, differential target multiplex stimulation modulated these pathways to a greater degree than conventional spinal cord stimulation. In this study, a total of 7,192 proteins could be identified, of which 1,451 were affected by differential target multiplex stimulation compared to only 705 that were affected by conventional stimulation [101].

## Conclusions

Pain medicine is a new, vitally important, but extremely challenging specialty. While pain is a nearly universal complaint, pain patients are a heterogeneous group. The medical approach to pain has sometimes been overly simplistic and prone to extremes, such as the American response to opioids, which ranged from overprescribing at first to underprescribing today. Old drug targets for pain are increasingly being supplanted by newer targets. In neuromodulation, the current generation of devices was used to electrically stimulate neurons without looking more deeply into the complexities of electrical signals on the central nervous system, including their stimulation of glial cells. Most pain therapies are prescribed to address a single type of pain mechanism, while pain is often mixed and requires multimodal approaches. Nevertheless, greater elucidation of pain, lessons learned, and a host of potential new therapeutic targets for pharmacological analgesics bring promise to pain management.
